# Mapping Myeloid Cell Diversity in Diffuse Large B-Cell Lymphoma: Impact on T Cell Exhaustion and Clinical Prognosis

**DOI:** 10.7150/jca.121954

**Published:** 2026-01-23

**Authors:** Jingwen Wang, Liangcheng Lv, Zhenjun Li, Xiaoyu Yao, Ning Ding

**Affiliations:** Key laboratory of Carcinogenesis and Translational Research (Ministry of Education), Laboratory of Lymphoma Translational Research, Peking University Cancer Hospital & Institute. No. 52 Fucheng Rd, Haidian District, Beijing, 100142, China.

**Keywords:** diffuse large B-cell lymphoma, tumor microenvironment, immune cell heterogeneity

## Abstract

Diffuse large B-cell lymphoma (DLBCL) is an aggressive subtype of B-cell lymphoma characterized by genetic variability and clinical heterogeneity. Single-cell sequencing technology enables mapping of intra-tumoral heterogeneity and microenvironment interactions. In this study, we analyzed single-cell and RNA expression microarray data from over 3,000 DLBCL patients to investigate the immune landscape of the tumor microenvironment and its association with clinical prognosis. Malignant B cells identified through B-cell receptor (BCR) clonal analysis and copy number variation (CNV) assessment exhibited enrichment in pathways related to the cell cycle, DNA replication and p53 signaling, which were closely related to adverse survival outcomes. Next, the myeloid cells derived from DLBCL tumor tissues could be further clustered into several distinct types, primarily comprising dendritic cells and macrophages. The increased prevalence of* SPP1*^+^ macrophages within the tumor microenvironment was correlated with inferior overall survival. Additionally, CellChat analysis revealed that frequent interactions between *SPP1*^+^ macrophages and CD8^+^ T cells may contribute to T cell exhaustion and create an immunosuppressive microenvironment. Collectively, the diverse sub-populations, particularly the immunosuppressive *SPP1*^+^ macrophages regulated immune suppression status within tumor microenvironment and represented a potential therapeutic target for DLBCL patients.

## Introduction

Diffuse large B-cell lymphoma (DLBCL) is the predominant subtype of B-cell lymphoma, accounting for approximately 30-40% of non-Hodgkin lymphomas (NHL)^1^,^2^. Despite advancements in clinical response and overall survival due to the incorporation of the anti-CD20 antibody rituximab alongside chemotherapy, approximately 30-40% of DLBCL patients continued to exhibit resistance to this combination immunotherapy^3^. Consequently, there is an urgent need to identify novel therapeutic strategies and molecular classifications of DLBCL to facilitate precise clinical management for these recurrent/refractory patients.

DLBCL can be categorized into two molecular subgroups, germinal center B-cell-like (GCB) and activated B-cell-like (ABC), using mRNA expression profiling (GEP)^4^. These subgroups showed different prognoses and responses to standard R-CHOP immunotherapy^5^. GCB-DLBCL and ABC-DLBCL made up about 40% and 50% of DLBCL cases respectively, with a small unclassifiable group comprising 10-15%^4^,^6^. Based on omics data analysis, the novel molecular DLBCL classification had recently been developed. The four genetic categories had been identified as MCD, BN2, N1, and EZB subtypes in DLBCL patients by oncogenic mutations, including *MYD88*, *CD79B*, *NOTCH1* and *NOTCH2* and *EZH2*, which improved the treatment strategy and prognosis of DLBCL patients^7^.

The biology and clinical behavior of DLBCL were influenced not only by the molecular alterations within the DLBCL tumor cells themselves but also by their interactions with the surrounding microenvironment. Evidence from both lymphoma patients and animal models suggested that within the lymphoma niche, external stimuli provided by microenvironmental cells and the extracellular matrix (ECM) played a significant role in disease development, progression, and response to treatment^8^-^10^. Consequently, elucidating the precise cellular and molecular mechanisms that facilitated tumor immune evasion remained a critical objective for enhancing current immunotherapies or developing novel therapeutic strategies. Based on the microenvironmental gene expression profiles, stromal-1 and stromal-2 signature had been identified to be related to the survival outcomes of DLBCL patients. The stromal-1 signature characterized by extracellular matrix deposition and histiocytic infiltration was associated with favorable survival in DLBCL. In contrast the stromal-2 signature marked by angiogenesis correlated with poor clinical outcomes^8^. Recently four distinct lymphoma microenvironment classification were found by transcription gene analysis, including “germinal center-like”, “mesenchymal”, “inflammatory” and “depleted” subtypes. Furthermore, patients with GC-like lymphoma microenvironment responded best to R-CHOP chemoimmunotherapy and had the highest survival rates, while those with “depleted” lymphoma microenvironment had the lowest response and survival rates, which indicated the significant prognostic value of tumor microenvronement^9^. Given the increasing evidence that the prognostic value of the TME was significant and independent of current standards, this area is of clear pathophysiological importance.

Given the controversies surrounding the latent role of the tumor microenvironment (TME) in the pathogenesis and progression as reported in several studies, recent research employing high-dimensional, single-cell analyses of primary tumors had significantly advanced our understanding of cancer biology^11^. The detailed characterization of cellular heterogeneity in DLBCL at the single-cell level hold promise for the development of more effective molecularly targeted therapies and prognostic biomarkers. In this study, we utilized single-cell RNA sequencing (scRNA-seq) and RNA expression microarray data from existing literature to construct a comprehensive cellular atlas of both malignant and non-malignant cells in DLBCL. This approach allowed us to investigate the impact of key signatures of malignant B cells and infiltrating immune cells on the pathogenesis and prognosis of DLBCL, potentially informing the development of novel molecularly targeted therapies and immunotherapeutic strategies.

## Results

### Generation of a single-cell atlas for DLBCL

**Figure 1:**
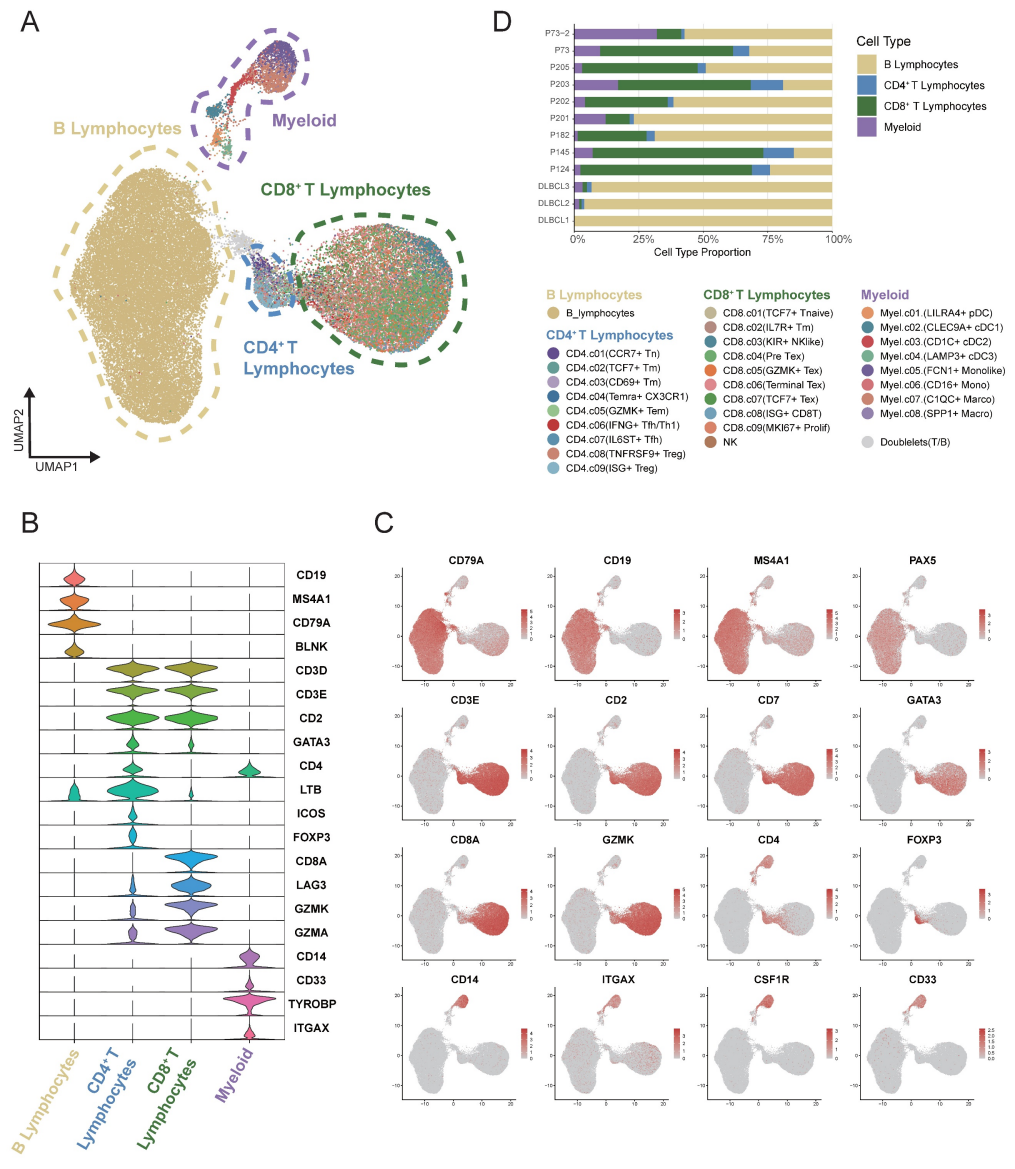
** Single-cell analysis revealed the heterogeneity of the tumor microenvironment in the integrated DLBCL samples.** (A) UMAP visualization of 54,199 single cells derived from DLBCL tumor tissues. UMAP plot showing the major lineages within tumor samples. Marker genes used for lineage definition were summarized in Supplementary Table S1. (B) Violin plots showing the expression of representative marker genes across major immune lineages. (C) UMAP plot showing the marker genes expression levels, with color intensity reflecting expression in individual cells. (D) Stacked bar plots showing the relative composition of immune cell subtypes across tumor samples from 12 patients, highlighting the inter-patient variability in microenvironmental cellular composition.

The single-cell RNA sequencing data derived from tumor tissues of 12 samples diagnosed with DLBCL (GSA-Human HRA002297 and GEO GSE182436) were reclustered and analyzed. A total of 54,199 high-quality single-cell transcriptomes from an initial 59,066 cells were analyzed, with an average of 2,378 genes detected per cell. T-cell receptor (TCR) and B-cell receptor (BCR) sequences were identified in 15,037 and 12,428 cells, respectively. Nonlinear dimensionality reduction was conducted using the Uniform Manifold Approximation and Projection (UMAP) method. Based on canonical marker gene expression, we discovered 4 different major clusters: B lymphocytes, myeloid cells, CD4^+^ T cells and CD8^+^ T cells (Figure 1A). The violin plot illustrated the principal genes for distinguishing the four major clusters (Figure 1B-C). B cells displayed a higher expression of *CD19*, *MS4A1* and *CD79A*. A higher expression of *CD33*, *TYROBP* and *ITGAX* was showed in myeloid cells. The expression level of *CD3D*, *CD3E*, and *CD4* was found to be markedly elevated in CD4^+^ T cells. *CD3D*, *CD3E* and *CD8A* genes were found to be highly expressed in CD8^+^ T cells. As illustrated in Figure 1D, the stacked bar chart depicted the four cell clusters in 12 DLBCL samples. Significant variation was observed in the proportions of these cell clusters among samples. B cells were the most abundant cell type presented in the DLBCL tumor tissues, with infiltrating T cells and myeloid cells being the next-most abundant.

### Heterogeneity of malignant B cell predicted the DLBCL prognosis

**Figure 2:**
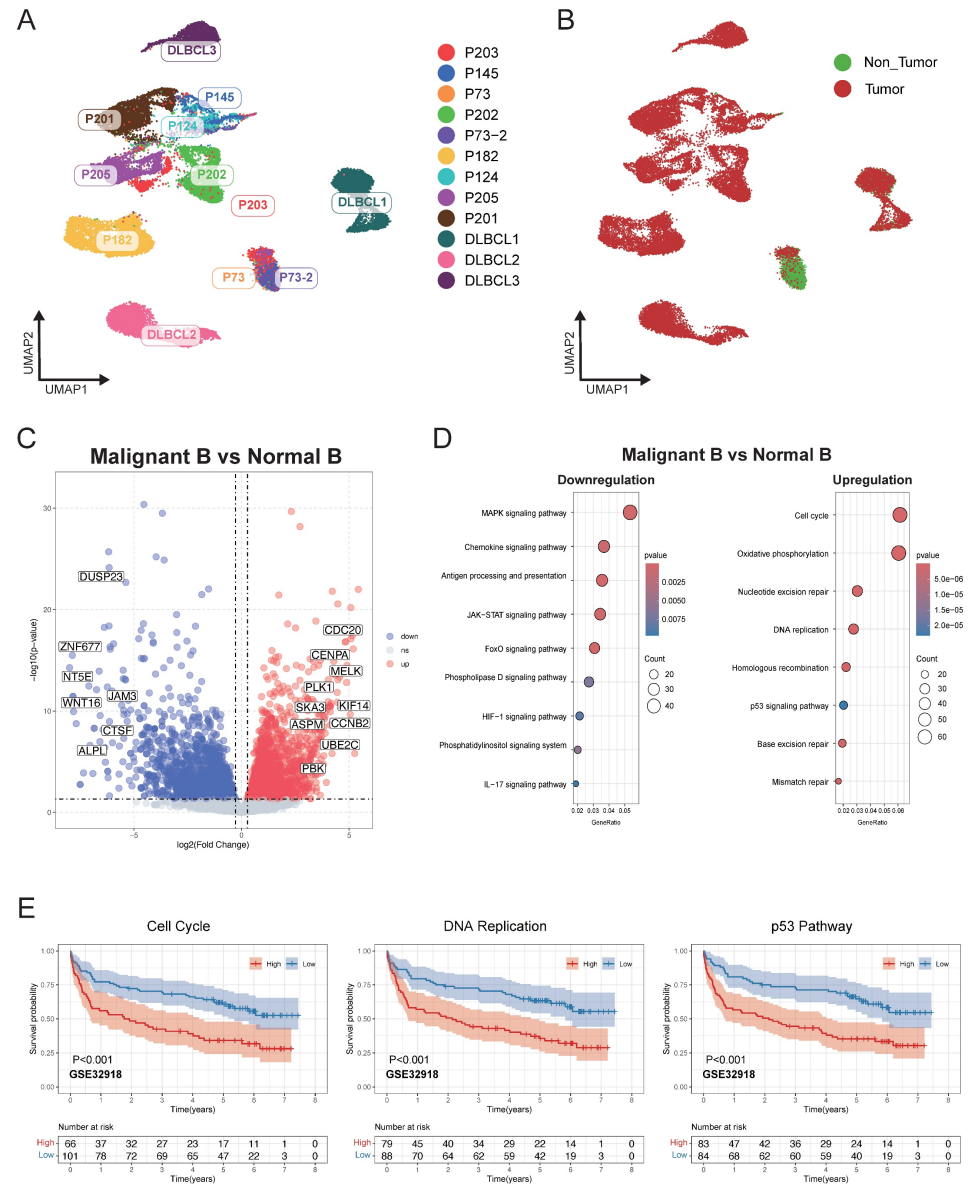
** Single-cell analysis revealed distinct transcriptional characteristics of malignant and non-malignant infiltrating B cells in DLBCL.** (A) UMAP plot showing B cells derived from 12 DLBCL patients. Each dot represents a single cell, colored by patient origin. (B) UMAP plot showing the malignant and non-malignant B cells. Malignant status was inferred using an integrated approach combining BCR clonality analysis and CNV estimation from scRNA-seq (inferCNV). (C) Volcano plot showing differentially expressed genes (DEGs) between malignant and non-malignant B cells. Significantly upregulated and downregulated genes were highlighted in red and blue, respectively (|log₂FC| > 1.5, p < 0.05). (D) Bubble plot presenting KEGG pathway enrichment analysis for the upregulated and downregulated DEGs. The size of the bubbles reflects the number of genes involved, while the color intensity represents the statistical significance of enrichment. (E) Kaplan-Meier survival curves illustrating the prognostic relevance of GSVA scores based on selected pathway signatures in external GEO RNA expression microarray datasets.

Malignant B cells and normal B cells were further identified from 12 DLBCL samples to study tumor heterogeneity in DLBCL. UMAP analysis was conducted to display the clustering of B cells belonging to each individual patient (Figure 2A). By using BCR clonal analysis and inferCNV assessment, we finally classified B cells into two clusters, consisting of 25,419 malignant B cells and 4,013 tumor-infiltrating normal B cells (Figure 2B, Figure S1A-E). Next, we made a comparison between the malignant and normal B cells of each sample to find significantly differentially expressed genes (Figure 2C). KEGG pathway analysis further revealed the significantly enriched pathways, including cell cycle, DNA replication, and p53 related signal pathways (Figure 2D). We validated the related pathway signatures using GEO cohorts. The Kaplan-Meier plot showed higher expression levels of genes associated with the cell cycle, DNA replication and p53 signal pathway were correlated with survival disadvantage within GEO database GSE32918 (Figure 2E), GSE181063 and GSE31312 (Figure S1).

### Clustering of myeloid cells from single-cell RNA sequencing

**Figure 3:**
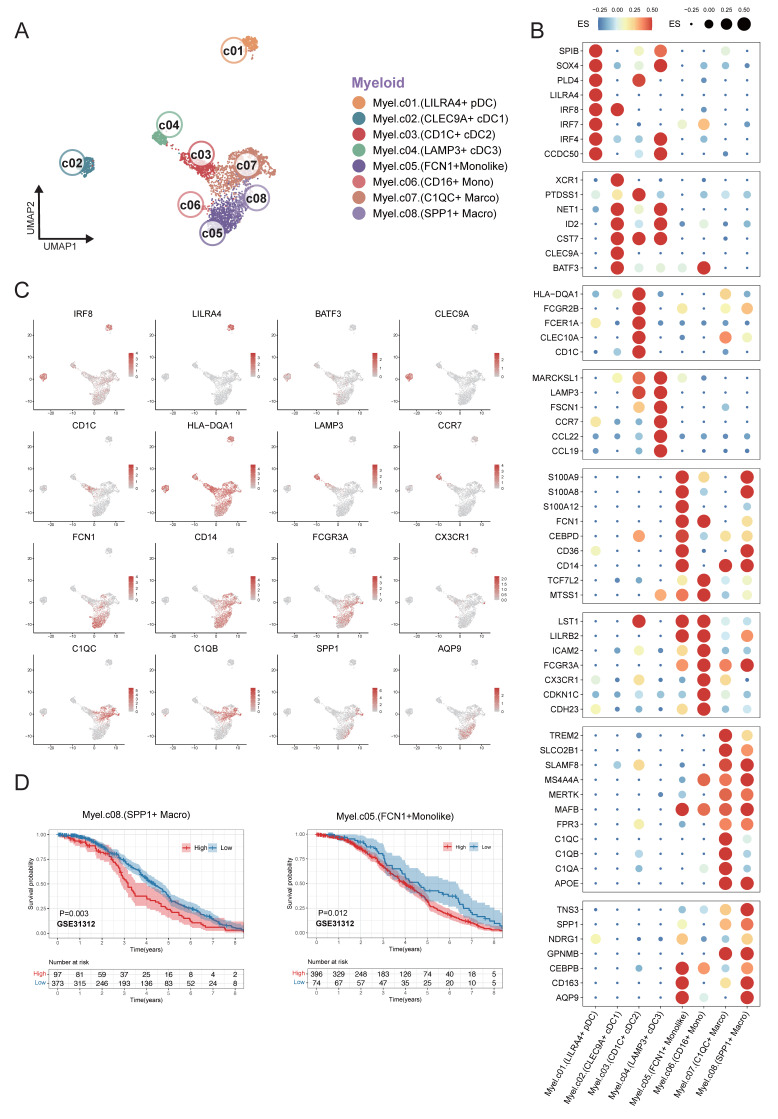
** Fine-grained characterization of myeloid cell subpopulations in DLBCL.** (A) UMAP plot showing the major lineages of myeloid cells. Marker genes used for lineage definition were summarized in Supplementary Table S1. (B) Dot plot showing the expression of marker genes associated with myeloid cells. Both dot size and color indicate effect size (ES) reflecting the average scaled expression level and the proportion of cells expressing each gene within a cluster. (C) UMAP plots colored by the log-normalized expression levels of selected canonical marker genes for myeloid cell subtypes based on scRNA-seq data. Color intensity reflects expression in individual cells. (D) Kaplan-Meier plot showing worse clinical outcome in DLBCL patients with the higher proportion of *SPP1*^+^ myeloid and *FCN1*^+^ moonlike cells in external GEO RNA expression microarray datasets. Patients were stratified based on enrichment scores of specific cell-type signatures, and differences in overall survival were assessed.

Eight myeloid cell subclusters were identified by single-cell RNA sequencing analysis within the integrated expression profiles of 12 DLBCL samples. This analysis characterized the transcriptional heterogeneity of infiltrating myeloid cells in DLBCL. The UMAP plot revealed distinct clustering of myeloid cells across patients based on their expression of immune markers (Figure 3A). We defined the identity of each cluster by evaluating subset-specific differentially expressed genes (Figure 3B-C). The *CLEC9A*^+^ cDC1 cluster was characterized by the expression of *CLEC9A*,* BATF3*, and* ID2*, whereas the *CD1C*^+^ cDC2 cluster was defined by *CD1C*, *FCGR2B*, and* CLEC10A*. The *LAMP*^+^ cDC3 cluster was distinguished by high expression levels of *LAMP3*, *FSCN1*, and *CCR7*. Clusters *C1QC*^+^ Macro and *SPP1*^+^ Macro could be differentiated based on the expression levels of *C1QA/B/C* and *SPP1* respectively. A subset of cells with high expression of *FCN1* was identified distinct from macrophages*,* which contained *FCN1*^+^ Monolike cells and *CD16*^+^ Monocytes. The *LILRA4*^+^ pDC cluster was defined by the expression of *LILRA4*,* IRF4*, and* IRF7*. Cluster distribution plot revealed that clusters *C1QC*^+^ Macro, *FCN1*^+^ Monolike, and *SPP1*^+^ Macro constituted the predominant populations across all samples, a finding that was corroborated by the UMAP analysis.

Based on clustering results from scRNA-seq data, we defined transcriptional signatures representative of distinct cell subpopulations. Subsequently, we evaluated the prognostic relevance between the signatures and patient survival outcomes using a cell type deconvolution algorithm in independent RNA expression microarray data cohorts from GEO database. There was a consistent indication that elevated levels of *FCN1*^+^ monolike cells and *SPP1*^+^ macrophages were associated with poor prognosis across multiple DLBCL cohorts GSE31312 (Figure 3D), GSE10846, GSE11318 and GSE87371 (Figure S2).

### Clustering of CD4^+^ T cells from single-cell RNA sequencing

**Figure 4:**
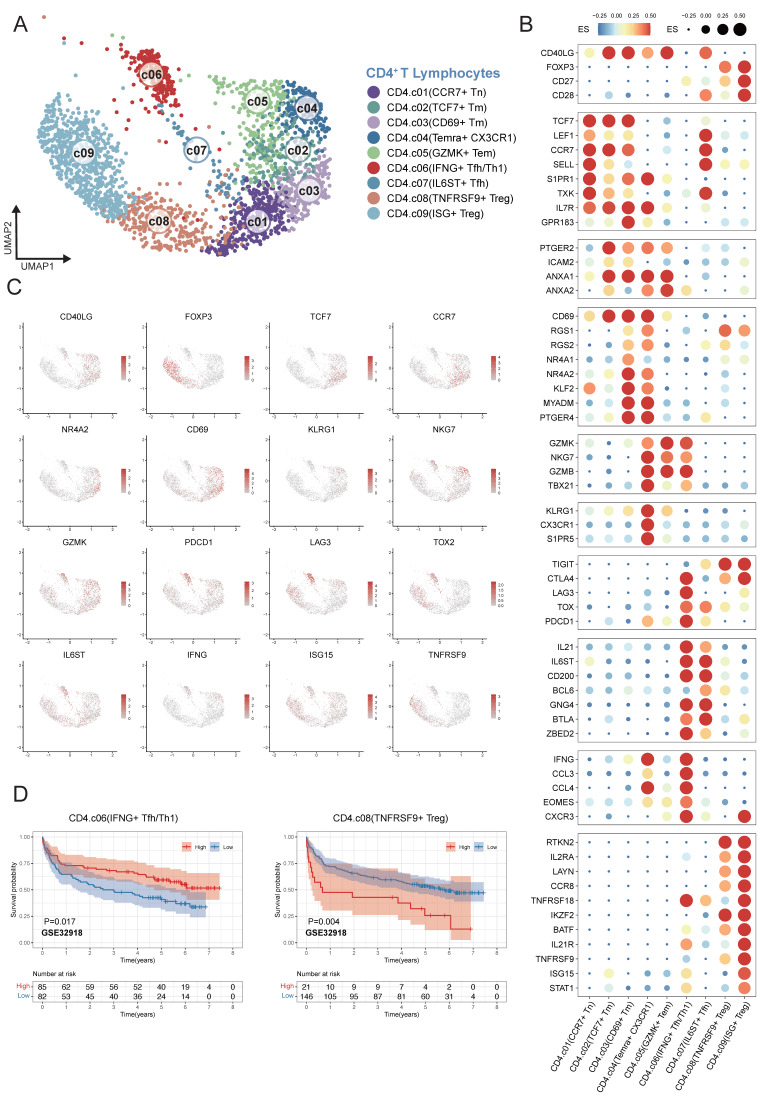
** Fine-grained delineation of CD4⁺ T-cell subpopulations in DLBCL.** (A) UMAP plot showing the major lineages of CD4^+^ T cells. Marker genes used for lineage definition were summarized in Supplementary Table S1. (B) Dot plot showing the expression of marker genes associated with CD4^+^ T cells. Both dot size and color indicate effect size (ES), reflecting the average scaled expression level and the proportion of cells expressing each gene within a cluster. (C) UMAP plots colored by the log-normalized expression levels of canonical CD4⁺ T-cell marker genes based on scRNA-seq data. Color intensity reflects expression in individual cells. (D) Kaplan-Meier survival curves demonstrating the prognostic relevance of selected signatures associated with CD4⁺ T-cell subsets in external GEO RNA expression microarray datasets. Patients were stratified based on enrichment scores of specific cell-type signatures, and differences in overall survival were assessed.

We characterized nine different CD4^+^ T cell clusters within the TME of DLBCL from single-cell transcriptomes in 12 samples (Figure 4A). In order to verify the reliability of the basis of nine clusters of CD4^+^ T cells, the expression patterns of representative marker genes in different cell clusters were analyzed. *CCR7*^+^ Tn cells were defined by the expression of *CCR7*, *SELL* and *S1PR1*; *TCF7*^+^ Tm cells by *TCF7*, *CCR7* and* IL7R*; *CD69*^+^ Tm cells by *CD69*, *NR4A1/2* and *MYADM*. Temra^+^ *CX3CR1* cells were characterized by *CX3CR1*, *KLRG1*, and *S1PR5*; *GZMK*^+^ Tem cells by *GZMK*, *GNLY*, and *NKG7*. *IFNG*^+^ Tfh/Th1 cells were marked by *IL21*, *IFNG*, and* CCL3*; whereas *IL6ST*^+^ Tfh cells expressed *IL6ST*, *CD200* and *BCL6*. *TNFRSF9*^+^ Treg cells were defined by *FOXP3* and *TNFRSF9*; ISG^+^ Treg mainly by interferon-stimulated genes (ISGs) (Figure 4B-C). To investigate the association between CD4⁺ T-cell subsets in tumor microenvironment and DLBCL patient's prognosis, we applied the same analytical framework used in myeloid cell analysis. Survival analyses revealed that an increased enrichment of *TNFRSF9*⁺ Treg cells within the TME was significantly associated with poor survival. Conversely, enriched proportion of *IFNG*⁺ Tfh/Th1 cells showed significant association with better outcomes across multiple independent GEO cohorts GSE32918 (Figure 4D) and GSE87371, GSE31312, GSE10846, GSE11318, GSE117556 (Figure S3).

### Clustering of CD8^+^ T cells from single-cell RNA sequencing

**Figure 5:**
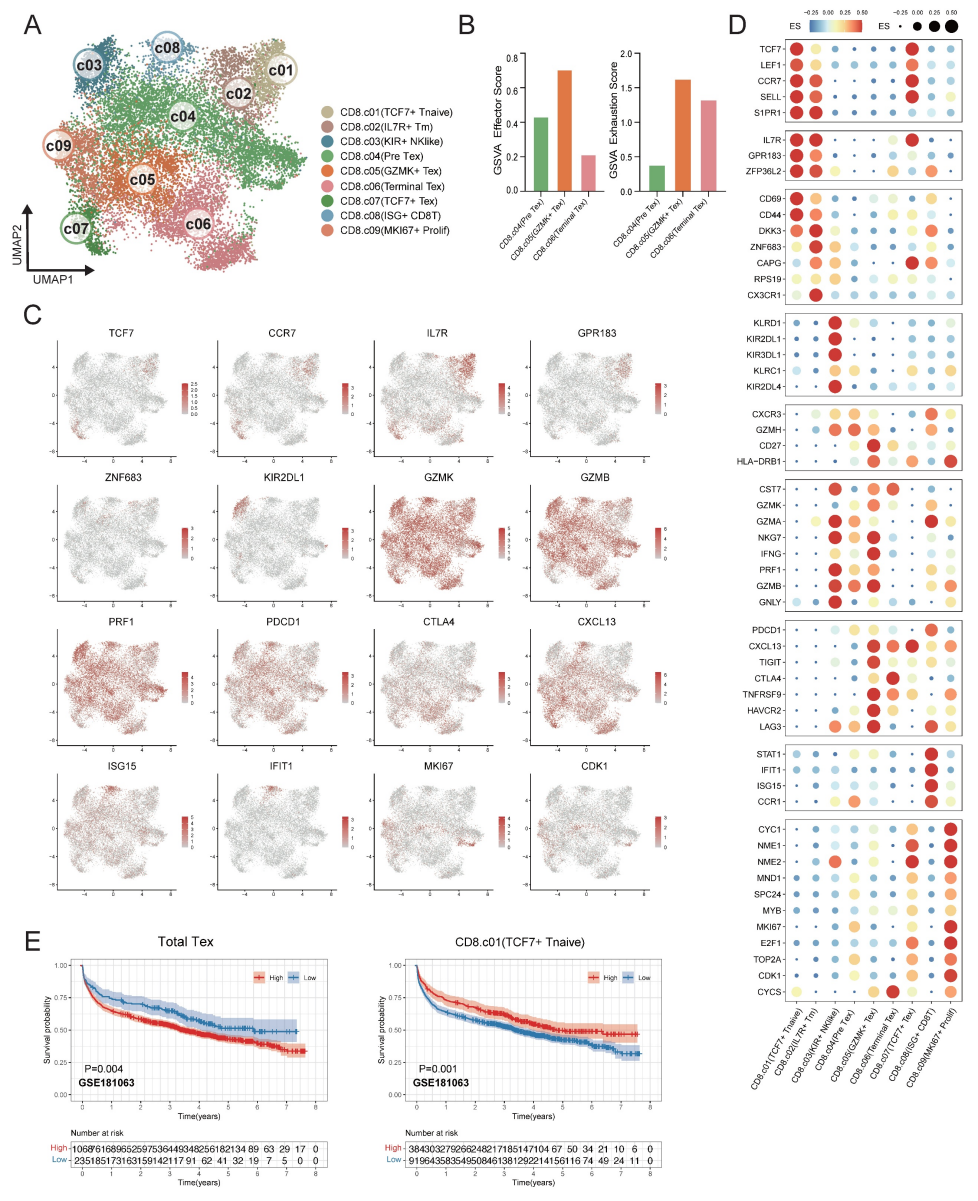
** Fine-scale characterization of CD8⁺ T-cell subpopulations in DLBCL.** (A) UMAP plot showing the major lineages of CD8^+^ T cells. Marker genes used for lineage definition are summarized in Supplementary Table S1. (B) Bar plots illustrating the GSVA scores for exhaustion and effector programs across Pre Tex, *GZMK*^+^ Tex and Terminal Tex cells. The exhaustion score was calculated based on the expression of *PDCD1*, *TOX*, *CXCL13*, *TIGIT*, *CTLA4*, *TNFRSF9*, *HAVCR2* and *LAG3*; the effector score was derived from *GNLY*, *GZMB*, *PRF1*, *IFNG*, *NKG7*, *GZMA*, *GZMK*, *CST7*, *TNF*, *FASL* and *TBX21*. (C) UMAP plots colored by log-normalized expression levels of selected canonical marker genes for CD8⁺ T-cell subsets based on scRNA-seq data. Color intensity reflects expression in individual cells. (D) Dot plot shows the expression of marker genes associated with CD8^+^ T cells. Both dot size and color indicate effect size (ES), reflecting the average scaled expression level and the proportion of cells expressing each gene within a cluster. (E) Kaplan-Meier survival curves evaluating the prognostic significance of selected CD8⁺ T-cell-related signatures in external GEO RNA expression microarray datasets. Patients were stratified based on enrichment scores of specific cell-type signatures, and differences in overall survival were assessed.

CD8⁺ T cells represent the predominant infiltrating lymphocyte population in the DLBCL tumor microenvironment. CD8⁺ T cells were analyzed and classified into nine distinct subpopulations based on their functional states and the expression profiles of lineage-defining genes across 12 samples (Figure 5A). To validate the reliability of this classification, we analyzed the expression patterns of representative marker genes across the identified clusters (Figure 5C-D). *TCF7*^+^ T Naïve cells were characterized by high expression of *TCF7* and *LEF1*, and *IL7R*^+^ Tm cells exhibited diminished *TCF7* expression alongside elevated *IL7R* and *ZNF683*, clearly distinguished from Naïve CD8⁺ T cells. *KIR^+^* NK like T cells were transcriptionally similar to NK cells and expressed *KIR* genes and *KLR* family genes. In the trajectory of CD8^+^ T cells, there was a transition from the pre-exhaustion T cells (Pre Tex), which expressed cytotoxic molecules, including *GZMK* and *GZMA* and low level of exhaustion markers, to cells *GZMK*^+^ Tex, which expressed inhibitory receptors such as *PDCD1* and *LAG3*. Next these cells changed into terminally exhausted T cells (Terminal Tex), which were characterized by high expression of exhaustion-related genes and reduced effector function (Figure 5B). The progression from Pre Tex cells to *GZMK*^+^ Tex, and finally to Terminal Tex cells indicated an important step of CD8⁺ T cells dysfunction in TME. Moreover, we observed a distinct subset of exhausted T cells (*TCF7*^+^ Tex) expressing exhaustion markers and stem-like gene *TCF7*, indicating a progenitor-exhausted signature. *ISG*^+^ CD8^+^ T cells showed increased expression of interferon-stimulated genes (*IFIT1* and *ISG15*). MKI67^+^ Prolif T cells showed increased *MKI67* and *NME1* expression.

To validate the association between CD8⁺ T cell subsets in the tumor microenvironment and clinical outcomes, survival analyses were conducted in the independent DLBCL cohorts. Remarkably, the DLBCL patients had a better prognosis with a higher proportion of *TCF7*^+^ T naïve cells. In contrast, the elevated proportion total Tex cells (combination of Pre Tex + *GZMK*^+^ Tex + Terminal Tex + *TCF7*^+^ Tex) were related to diminished survival rate in DLBCL patients within GSE181063 (Figure 5E), GSE11318, GSE117556, GSE32918, GSE10846 and GSE87371 (Figure S4).

### Interaction between SPP1^+^ macrophages and CD8^+^ cells

**Figure 6:**
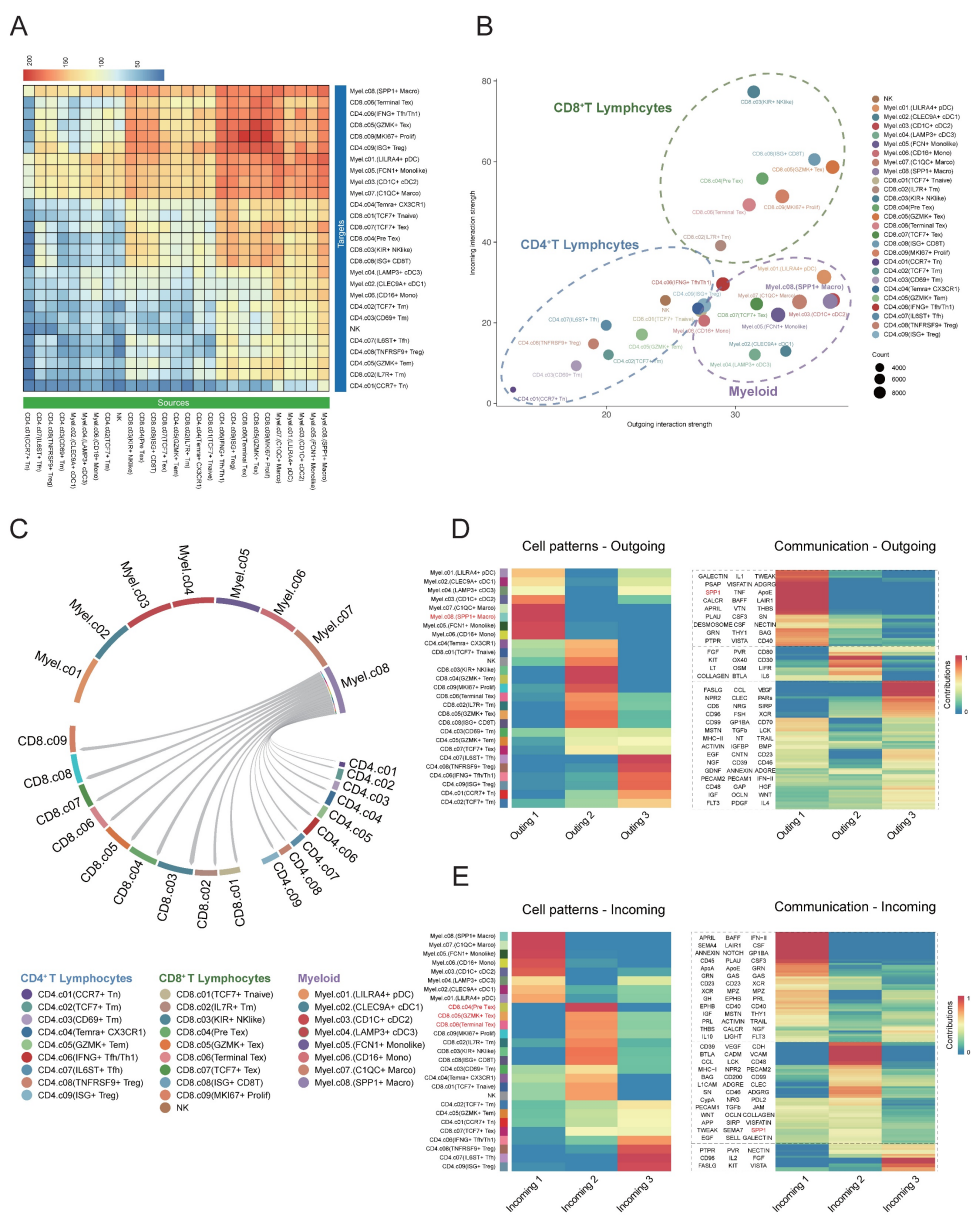
** Cell-cell communication between SPP1⁺ cells and CD8⁺ T cells in the DLBCL tumor microenvironment.** (A) Heatmap showing the inferred cell-cell communication events between different subclusters. Columns represent signal-sending cells, and rows represent signal-receiving cells. The color represented the strength of interaction. (B) Scatter plot depicting cell-cell communication network centrality. The X-axis represents the contribution of each cell type in transmitting signals, while the Y-axis indicates its role in receiving signals. (C) Circular chord diagram illustrating interaction strength between* SPP1*⁺ Macro cells and T cells. Each ribbon represents a communication event, with *SPP1*⁺ Macro cells as signal sources and T cells as recipients. (D) Left: Heatmap of NMF-based clustering of cells according to outgoing signaling patterns. Color intensity reflects the relative contribution of each cell type to the corresponding signaling module. Right: Heatmap showing the composition of signaling pathways within each NMF-defined module. Color intensity denotes the strength of intercellular communication for each pathway. (E) Heatmap as in (D) but based on incoming signaling patterns.

Subsequently, we employed CellChat to analyze intercellular communication within the DLBCL tumor microenvironment focusing on ligand-receptor interactions and associated signaling pathway. The heatmap plot showed that the major regulatory axis in the TME was the infiltrating CD8^+^ T lymphocytes and myeloid cells (Figure 6A). The network centrality analysis further clarified the functional roles of various cell types in mediating cell communications. Within the network of the TME, CD8⁺ T cells occupied a central hub position and exhibited significant signal senders' and receivers' potential. Myeloid cells mainly acted as robust signal initiators with limited ability to receive signals, suggesting they might function as important upstream regulators in the immune signaling cascade (Figure 6B). Notably, the *SPP1*⁺ macrophages may play an important regulatory role in the DLBCL TME due to their remarkably strong signal sending capacity.

The study also identified that *SPP1⁺* macrophages were widespread communicators to all clusters of CD8⁺ T cells. Early CD8⁺ T cells, such as CD8⁺ naïve and Pre Tex cells, showed limited communication efficacy with *SPP1*⁺ macrophages. On the other hand, the activated CD8^+^ subsets, including *KIR*^+^ NK like CD8+ T cells and *GZMK*^+^ Tex cells exhibited increased incoming signals from *SPP1*⁺ macrophages (Figure 6C). As indicated by these findings, *SPP1*⁺ macrophages may interact preferentially with activated CD8⁺ T cells which may alter their functional states thus remodeling DLBCL tumor microenvironment.

We carried out non-negative matrix factorization analysis (NMF) to identify key outgoing (sending) and incoming (receiving) signaling and key ligand-receptor interactions patterns. The heatmap visualization showed the contributions of various cell types to different signaling. Myeloid cells especially the *SPP1*^+^ macrophages exhibited strong signal-sending capacities within the outgoing signaling heatmap. Conversely, the incoming signaling map indicated that CD8^+^ T cells, especially Pre-Tex, *GZMK*^+^ Tex and Terminal Tex subsets, exhibited significant signal-receiving activity. Meanwhile, pathway enrichment analysis showed that the SPP1 ligand-receptor axis was largely responsible for these interactions (Figure 6D-E). The data implied that myeloid cell clusters, specifically *SPP1*^+^ macrophages, may facilitate the shift of CD8^+^ T cells from the effector state toward terminal exhaustion while contributing to the remodeling of the tumor microenvironment and immune suppression.

## Discussion

In our study, we analyzed single-cell and RNA expression microarray data to reveal the possible relationship between the immune landscape transcriptome and clinical outcomes in DLBCL patients. We created a comprehensive cell atlas encompassing both malignant and nonmalignant cell populations, providing evidence that the diversity of myeloid and T lymphocyte subpopulations, along with specific transcriptomic gene signatures, significantly predicted the prognosis of DLBCL patients.

Secreted Phosphoprotein-1 (SPP1), also known as Osteopontin (OPN) was extensively expressed across a variety of cell types, including T cells, B cells and myeloid cells^12^. Additionally, SPP1 was found in body fluids such as serum, bovine milk, and human urine, where it played a role in intercellular communication and the extracellular matrix^13^. Elevated levels of circulating SPP1 in serum, as well as increased SPP1 expression in tumor cells, had been associated with poor prognosis in multiple cancer types by promoting tumor cell proliferation, migration, and invasion^14^,^15^. Regarding circulating SPP1, the expression level of SPP1 in tumor tissue did not correlate with plasma SPP1 levels and patient outcomes, suggesting that non-malignant cells may contribute to plasma SPP1 concentrations^16^. Recently, the expression of SPP1 by macrophages had garnered significant attention in the scientific community. Eri Matsubara and colleagues have reported that elevated SPP1 expression in tumor-associated macrophages (TAMs) was indicative of a poor prognosis in patients with lung adenocarcinoma^17^. More recently, Ruben Bill et al. demonstrated that SPP1 played a role in the progression of macrophage polarization, with IFN-γ-induced CXCL9 and hypoxia-induced SPP1 emerging as critical features of the tumor microenvironment^18^. Furthermore, the knockdown of SPP1 in macrophages had been shown to mitigate tumor cell migration and induce the Th1 response by downregulating PD-L1^19^. Our findings revealed that *SPP1*^+^ macrophages fostered an immunosuppressive microenvironment in DLBCL by suppressing T-cell activation and effector function, consistent with the finding that SPP1 promotes metastatic tumor growth in non-small-cell lung cancer^20^. Importantly, *in vivo* administration of the SPP1-blocking antibody suppressed liver and lung metastases^21^. Thus, the abundance of *SPP1*^+^ TAMs may serve as a predictive biomarker for patient stratification and as a therapeutical target to overcome immunotherapy resistance in DLBCL. Future studies integrating SPP1 pathway may constitute a rational and promising strategy to restore anti-tumor immunity and improve outcomes in patients with refractory disease.

The complement system, a critical component of the innate immune system, was instrumental in the recognition and elimination of pathogens^22^. Numerous studies had demonstrated that the expression of C1q was correlated with markers indicative of M2-like macrophage phenotypes. Zhang et al. identified that C1q-positive TAMs exhibited high expression levels of several macrophage markers, including *CD163* and *APOE*, as well as inhibitory molecules such as *Tim-3* and *PD-1*, suggesting an immunosuppressive role within the tumor microenvironment^23^. Furthermore, research by Lubka Roumenina et al. indicated that TAMs were the predominant cell type responsible for C1q production, and the density of C1q-positive cells were associated with poor prognosis in advanced clear cell renal cell carcinoma (ccRCC)^24^. Additionally, C1q-expressing TAMs and complement activation products were found to promote inflammation, T-cell exhaustion, and tumor progression. Notably, the ablation of C1q, C4, and C3 in mice resulted in a reduction in tumor growth^24^.

T cell exhaustion referred to a range of dysfunctional states observed in antigen-specific CD8^+^ T cells, a concept initially characterized in the context of chronic viral infections^25^. Due to mechanisms of immunotolerance and immunosuppression, CD8^+^ T cells infiltrating tumors often failed to achieve full activation, subsequently transitioning into an exhausted and dysfunctional state characterized by diminished proliferative capacity, reduced cytokine production, and impaired tumor cell lysis^26^. Gene expression profiling had demonstrated that exhausted T cells exhibited upregulation of immune checkpoint receptors, including PD-1, CTLA-4, Tim-3, LAG-3^27^-^29^. The therapeutic targeting of these inhibitory receptors, particularly CTLA-4 and PD-1, with specific antibodies had led to significant improvements in clinical outcomes for patients with advanced melanoma, non-small-cell lung cancer, renal cell carcinoma, and B cell malignancies^30^-^33^. Numerous studies have indicated that the extent of exhausted T-cell infiltration correlates with the response rates to immunotherapy. Similarly, in our study, DLBCL patients with a higher percentage of total exhausted CD8^+^ T cells within tumor microenvironment tended to have worse survival outcomes. More importantly, *TCF7* expression regulated differentiation of T cells in tumor tissue^34^. The loss of T-cell exhaustion regulatory genes, such as *NR4a1* and *NR4a2*, triggered *TCF7* expression promoted anti-tumor immunity in tumor infiltration CD8^+^ T cells 35. Similarly, the infiltration of stem-like exhausted T precursor/progenitor cells, such as TCF7^+^ Tex cells, were correlated with functionality of memory T cells and favorable prognosis of DLBCL patients.

The sequence of processes facilitating DNA replication and cell division was referred to as the cell cycle. The advancement of cancer was characterized by aberrant activity within the cell cycle^36^. DNA mutations contributed to the dysregulation of cell cycle kinases; a phenomenon frequently associated with abnormal cell division and the uncontrolled proliferation characteristic of cancer cells. Notably, aberrant activation of cyclin-dependent kinases (CDKs), a common occurrence in human malignancies, underscored the rationale for developing synthetic CDK inhibitors as anticancer therapeutics^37^. Concerning the molecular signatures within DLBCL tumor cells, our results illustrated that cell cycle, DNA replication, and p53 signal pathway related genes were significantly upregulated in malignant B cells. Consistent with our results, the upregulated cyclins, cell cycle-specific antigens PCNA and mini-chromosome maintenance (MCM) proteins promoted the progression of cell cycle regulation, DNA replication in various kinds of tumor development 38-40.

In summary, the intra-tumoral heterogeneity and microenvironmental interactions characteristic of DLBCL present promising opportunities for prognostic stratification and the advancement of novel immunotherapeutic strategies.

## Material and Methods

### Data process and single cell sequence analysis

Two DLBCL scRNA-seq datasets were sourced from GSA-Human (HRA002297)^41^ and GEO (GSE182436)^10^. The HRA002297 dataset comprises single-cell data derived from nine DLBCL tumor tissue samples, while the GSE182436 dataset included data from three patients. After merging the datasets, we utilized Seurat (v5.2.1) to process the Unique Molecular Identifier (UMI) count matrices which represent the number of unique transcripts captured per cell and conducted quality control, keeping 54,199 cells and 41,166 genes for subsequent analysis^42^,^43^. The scRNA-seq data was processed through log transformation, normalization, and dimensionality reduction, followed by visualization. The Variance Stabilizing Transformation (VST) method pinpointed the top 2,000 highly variable genes, and Principal Component Analysis (PCA) was used for dimensionality reduction, with UMAP applied for clustering and visualization^44^. Batch effects were corrected using Harmony Integration in Seurat. The Leiden algorithm was employed for multiscale clustering to identify cell meta clusters, which were annotated based on gene expression profiles^45^. Myeloid cells, CD4⁺ T cells, and CD8⁺ T cells were then analyzed using the same preprocessing and clustering approach. The related marker genes were chosen according to canonical immune cell signatures reported in prior single-cell studies of the tumor microenvironment and hematopoietic lineages (Supplementary Table S1) 46-48. Concerning the microarray data, we selected seven independent DLBCL RNA expression microarray data cohorts from the Gene Expression Omnibus (GEO) database (GSE10846, GSE11318, GSE31312, GSE32918, GSE87371, GSE117556 and GSE181063), which totally contained clinical information and expression data from over 3,000 DLBCL patients.

### Malignancy Identification

First, we analyzed BCR sequencing data and identified dominant monoclonal and polyclonal B cell subsets. Subsequently, we performed single-cell Copy Number Variation (CNV) analysis for these subsets using the InferCNV (v1.22.0) algorithm. The malignant B cells were identified based on BCR clonality analysis, κ/λ chain ratio and InferCNV results^49^. The reference population for InferCNV was selected from a cluster containing cells derived from multiple patient samples that showed no detectable copy number alterations, supporting its suitability as a non-malignant baseline.

### Cellular composition deconvolution and survival analysis

Seven cohort datasets were obtained from the GEO database, which contained over 3,000 samples and survival data of patients. We utilized scRNA-seq clustering to determine a transcriptional signature for specific cell subpopulations. Next, we used the BayesPrism algorithm to establish a prognosis model using risk scores generated from these datasets to determine its relationship with patients' survival outcome^49^. The Overall Survival (OS) was defined as the time from initiation of disease until death from any cause. Survival R package (Version 3.8-3) was employed to study the correlation of risk factors with survival probability^50^. Univariate and multivariate Cox regression analyses were conducted, presented with 95% confidence intervals, to assess the effect of the mentioned factors on survival endpoints. Results shown statistically significant if p value < 0.05. We only considered findings that showed consistent survival trends as well as statistical significance in at least three studies as clinically relevant.

### Cell-cell communication analysis

CellChat (version 2.1.2) was used to analyze intercellular communication based on a curated ligand-receptor interactions^51^. The analysis was performed on the basis of gene expression data, quantified as transcriptome-wide read counts mapped to protein-coding genes. The average expression level for each identified cell cluster was computed and served as input to infer communication probabilities and reconstruct signaling networks.

### KEGG pathway enrichment analysis

Pseudobulk count matrices for each cell type were generated from the Seurat object and normalized using DESeq2. Principal Component Analysis (PCA) was performed to evaluate sample reproducibility and the stability of inter-sample variation. Differentially Expressed Genes (DEGs) were identified using a threshold of |log₂ fold change| > 1.5 and p < 0.05, and visualized with volcano plots generated by ggplot2. KEGG pathway enrichment analysis of DEGs was conducted using cluster Profiler (v4.14.4), and the top pathways were ranked by p-values. Gene Set Variation Analysis (GSVA) (v2.0.5) was performed on selected gene sets or pathways to calculate enrichment scores for further analysis (Supplementary Table S2).

## Supplementary Material

Supplementary figures and tables.

## Abbreviations

DLBCL: diffuse large B-cell lymphoma
BCR: B-cell receptor
CNV: copy number variation
NHL: non-Hodgkin lymphomas
GCB: B-cell-like
ABC: activated B-cell-like
GEP: mRNA expression profiling
ECM: extracellular matrix
TME: tumor microenvironment
scRNA-seq: single-cell RNA sequencing
TCR: T-cell receptor
UMAP: uniform manifold approximation and projection
ISGs: interferon-stimulated genes
Pre Tex: pre-exhaustion T cells
Terminal Tex: terminally exhausted T cells
SPP1: secreted phosphoprotein-1
OPN: osteopontin
TAMs: tumor-associated macrophages
ccRCC: clear cell renal cell carcinoma
CDKs: cyclin-dependent kinases
MCM: mini-chromosome maintenance
VST: variance stabilizing transformation
PCA: principal component analysis
GEO: gene expression omnibus
OS: overall survival
DEGs: differentially expressed genes
GSVA: gene set variation analysis
UMI: unique molecular identifier
