# Supplementary material for: Mapping Myeloid Cell Diversity in Diffuse Large B-Cell Lymphoma: Impact on T Cell Exhaustion and Clinical Prognosis
Source: J Cancer. 2026 Jan 23;17(3):469–82. doi: 10.7150/jca.121954 (PMC13003549; doi:10.7150/jca.121954)
Supplement: Supplementary file 1 — Supplementary figures and tables. [file jcav17p0469s1.pdf]

Figure S1

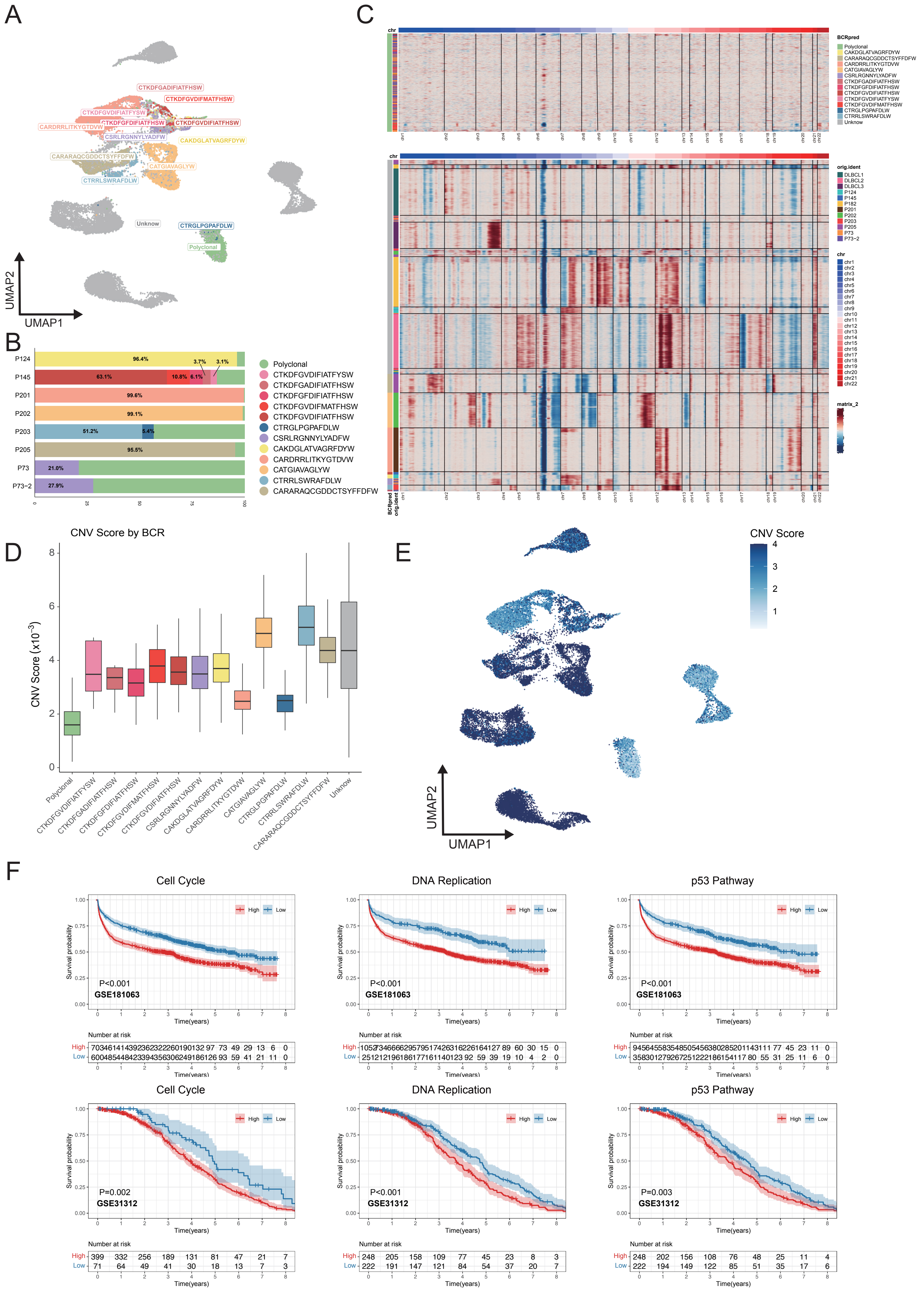

A
CTKDFGADIFIATFHSW
CTKDFGVDIFMATFHSW
CTKDFGVDIFIATFYSW
CTKDFGFDIFIATFHSW
CTKDFGVDIFIATFHSW
CARDRRLITKYGTDVW
CSRLRGNNYLYADFW
CAKDGLATVAGRFDYW
CARARAQCGDDCTSYFFDFW
CATGIAVAGLYW
CTRRLSWRAFDLW
Unknow
CTRGLPGPAFDLW
Polyclonal
UMAP2
UMAP1
B
P124
P145
P201
P202
P203
P205
P73
P73−2
96.4%
3.7%
3.1%
63.1%
10.8%
6.1%
99.6%
99.1%
51.2%
5.4%
95.5%
21.0%
27.9%
0
25
50
75
100
Polyclonal
CTKDFGVDIFIATFYSW
CTKDFGADIFIATFHSW
CTKDFGFDIFIATFHSW
CTKDFGVDIFMATFHSW
CTKDFGVDIFIATFHSW
CTRGLPGPAFDLW
CSRLRGNNYLYADFW
CAKDGLATVAGRFDYW
CARDRRLITKYGTDVW
CATGIAVAGLYW
CTRRLSWRAFDLW
CARARAQCGDDCTSYFFDFW
C
chr
BCRpred
orig.ident
matrix_2
DLBCL1
DLBCL2
DLBCL3
P124
P145
P182
P201
P202
P203
P205
P73
P73−2
D
CNV Score by BCR
CNV Score (x10^{-3})
E
CNV Score
UMAP2
UMAP1
F
Cell Cycle
DNA Replication
p53 Pathway
High
Low
Survival probability
Time(years)
P<0.001
GSE181063
Number at risk
P=0.002
P=0.003
GSE31312

Figure S1.

(A) UMAP plot showing the distribution of BCR dominant monoclonal populations across different patients. Each point represented a single cell and was clustered by its corresponding BCR clonotype. Cells lacking BCR sequencing data were labeled as "unknown" and shown in grey. (B) Stacked bar plot showing the proportions of dominant monoclonal BCR clonotypes. (C) Heatmap showing InferCNV analysis results. The CNV analysis was performed in these subsets with and without BCR sequencing information at the single-cell level using the InferCNV algorithm. The reference population showed no obvious copy number variation. (D) CNV scores calculated by BCR clonotype subtype. (E) UMAP plot showing the distribution of CNV scores across individual cells. (F) Supplementary Kaplan–Meier survival curves among DLBCL patient groups stratified by GSVA scores of selected pathways.

Figure S2

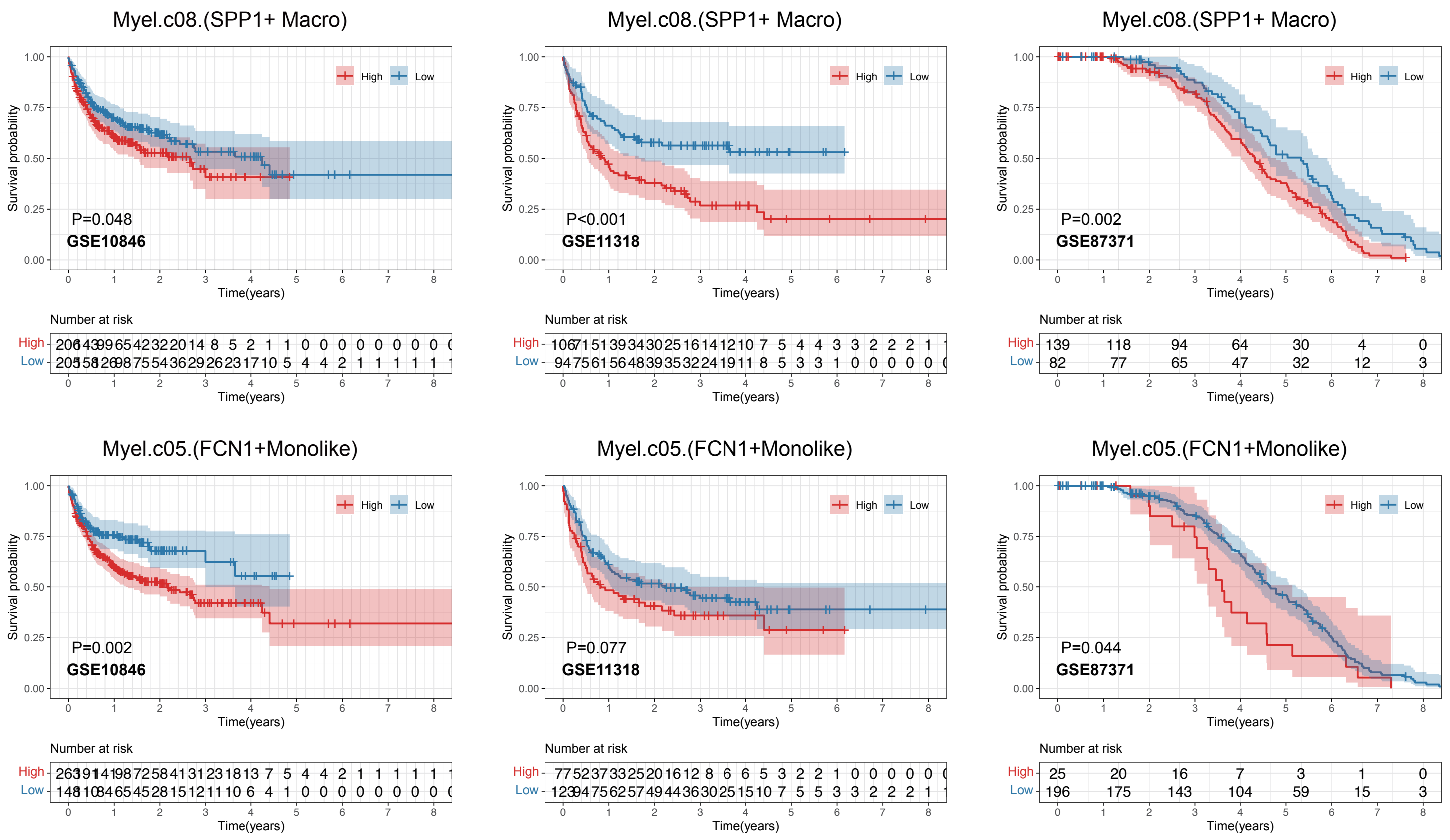
Myel.c08.(SPP1+ Macro)
High
Low
Survival probability
Time(years)
P=0.048
GSE10846
Number at risk
Myel.c08.(SPP1+ Macro)
P<0.001
GSE11318
Myel.c08.(SPP1+ Macro)
P=0.002
GSE87371
High 139 118 94 64 30 4 0
Low 82 77 65 47 32 12 3
Myel.c05.(FCN1+Monolike)
P=0.002
GSE10846
Myel.c05.(FCN1+Monolike)
P=0.077
GSE11318
Myel.c05.(FCN1+Monolike)
P=0.044
GSE87371
High 25 20 16 7 3 1 0
Low 196 175 143 104 59 15 3

Figure S2.

Supplementary Kaplan–Meier survival curves among DLBCL patient groups stratified by the enrichment levels of *FCN1*$^{+}$ monolike cells and *SPP1*$^{+}$ macrophages.

Figure S3

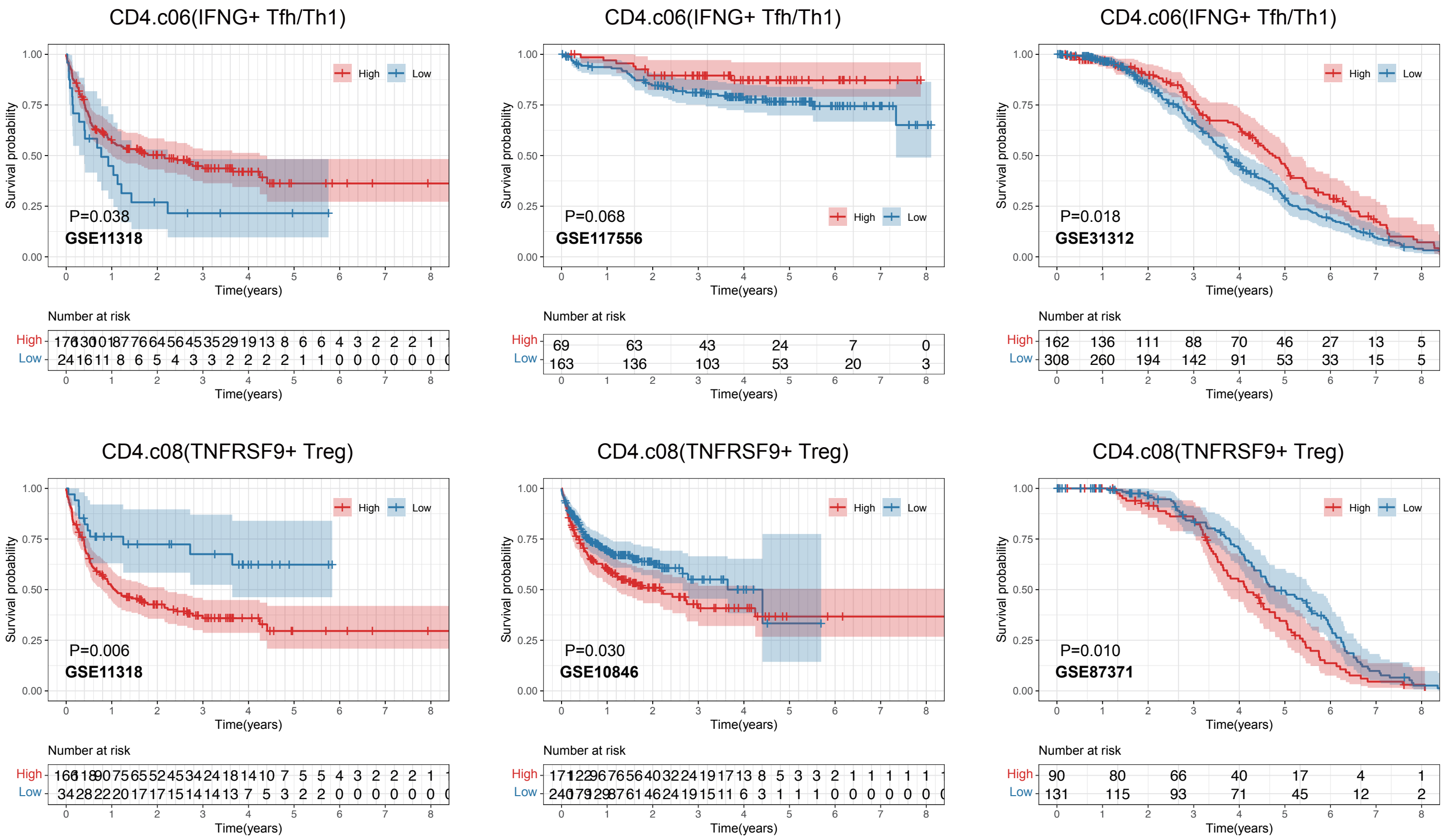

CD4.c06(IFNG+ Tfh/Th1)
P=0.038
GSE11318
CD4.c06(IFNG+ Tfh/Th1)
P=0.068
GSE117556
CD4.c06(IFNG+ Tfh/Th1)
P=0.018
GSE31312
CD4.c08(TNFRSF9+ Treg)
P=0.006
GSE11318
CD4.c08(TNFRSF9+ Treg)
P=0.030
GSE10846
CD4.c08(TNFRSF9+ Treg)
P=0.010
GSE87371
High
Low
Survival probability
Time(years)
Number at risk

Figure S3.

Supplementary Kaplan–Meier survival curves among DLBCL patient groups stratified by the enrichment levels of *TNFRSF9*$^{+}$ Treg cells and *IFNG*+ Tfh/Th1 T cells.

Figure S4

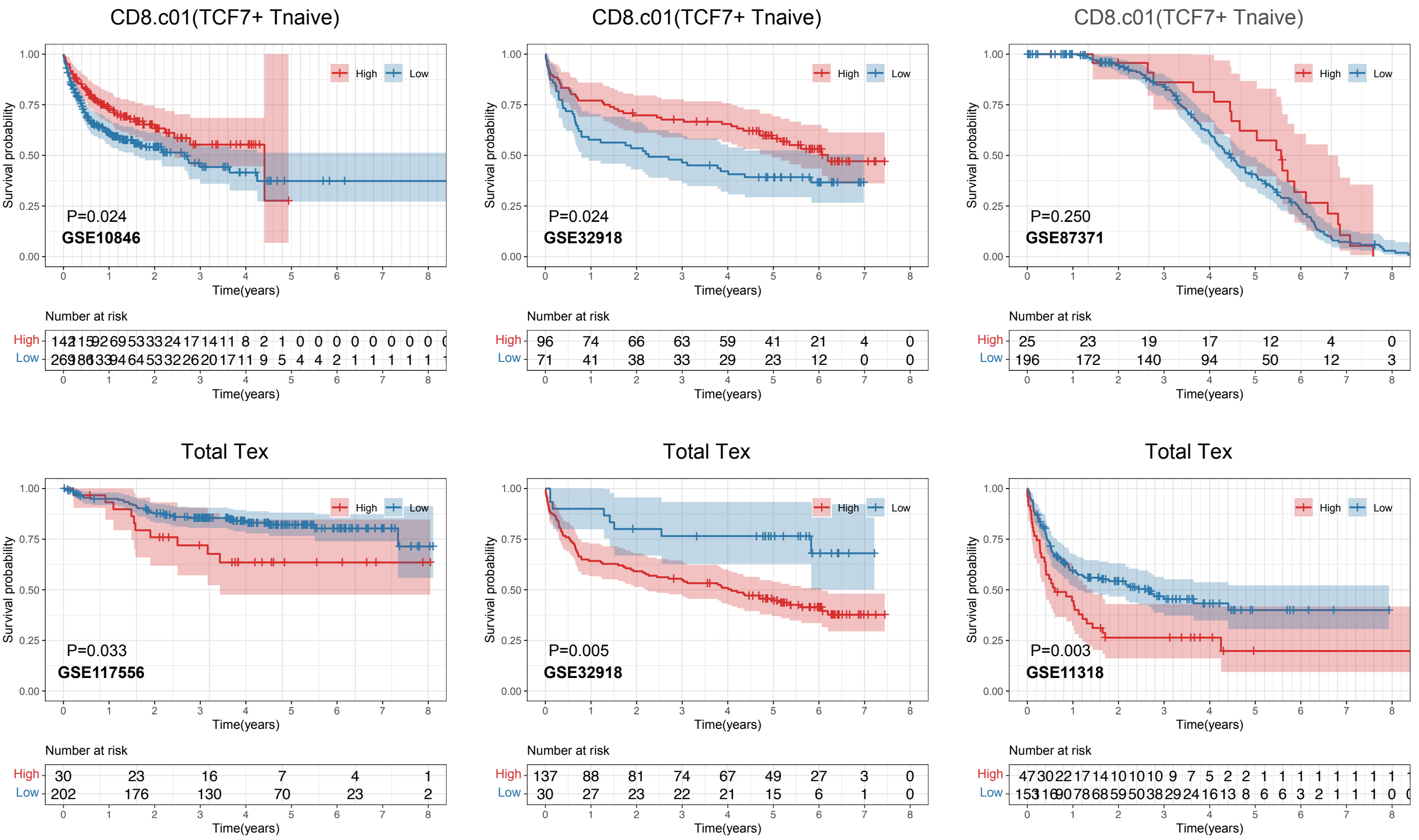
CD8.c01(TCF7+ Tnaive)
P=0.024
GSE10846
P=0.024
GSE32918
P=0.250
GSE87371
Total Tex
P=0.033
GSE117556
P=0.005
GSE32918
P=0.003
GSE11318
Survival probability
Time(years)
High
Low
Number at risk

Figure S4.

Supplementary Kaplan–Meier survival curves among DLBCL patient groups stratified by the enrichment levels of Total Tex cells and *TCF7*$^{+}$ naïve CD8 cells.

Table S1. Signature Genes for All Cell Subsets. Related to Figure 1, 3, 4 and 5.

Note that this table contains the following worksheets:	
**Worksheet**	**Title**
A	Signature Genes Used to Define B cells, Myeloid cells, CD4+T cells and CD8+T cells.
B	Signature Genes Used to Define Myeloid cells subsets.
C	Signature Genes Used to Define CD4+ T cells subsets.
D	Signature Genes Used to Define CD8+ T cells subsets.

Table S1A. Signature Genes Used to Define B cells, Myeloid cells, CD4+T cells and CD8+T cells. Related to Figure 1.

B	Myeloid	CD4+T	CD8+T
CD19	CD14	CD3D	CD3D
MS4A1	ITGAX	CD3E	CD3E
CD79A	TYROBP	CD2	CD2
BLNK	CD33	CD4	CD8A
		LTB	LAG3
		GATA3	GZMK
		ICOS	GZMA
		FOXP3	

Table S1B. Signature Genes Used to Define Myeloid cells subsets. Related to Figure 3.

Myel.c01.(LILRA4+ pDC)	Myel.c02.(CLEC9A+ cDC1)	Myel.c03.(CD1C+ cDC2)	Myel.c04.(LAMP3+ cDC3)	Myel.c05.(FCN1+ Monolike)	Myel.c06.(CD16+ Mono)	Myel.c07.(C1QC+ Marco)	Myel.c08.(SPP1+ Macro)
CCDC50	ANPEP	ADAM28	CCL19	CD14	CDH23	ACP5	ABL2
GAS6	ANXA6	ADAM8	CCL22	CD36	CDKN1C	APOC1	ADM
GZMB	BATF3	ARAF	CCR7	CEBPD	CX3CR1	APOE	AQP9
IL3RA	C1ORF54	AXL	FSCN1	CXCR4	FCGR3A	ATF3	C15ORF48
IRF4	CCR7	CD1A	LAMP3	FCGR2A	FCGR3B	C1QA	CD163
IRF7	CD40	CD1C	MARCKSL1	FCGR3A	FCN1	C1QB	CEBPB
IRF8	CLEC9A	CD1E		FCN1	FLI1	C1QC	CSTB
ITM2C	CLIC2	CLEC10A		HLA-DQA1	ICAM2	CD81	CXCL2
JCHAIN	CST7	FCER1A		HLA-DQB1	IFITM1	CSF1R	EREG
LILRA4	DAPP1	FCGR2B		HSPA1A	IFITM2	ENG	FN1
LILRB4	ETV3	HLA-DQA1		HSPA1B	IKZF1	FCGR1A	GPNMB
LY9	FLT3	HMGA1		MAFB	KLF3	FPR3	IL1RN
MZB1	ID2	IRF4		MTSS1	LILRA	MAF	MARCO
PLAC8	IDO1	LY86		S100A12	LILRB1	MAFB	NDRG1
PLD4	IL6ST	PFDN1		S100A8	LILRB2	MEF2A	OLR1
SELL	NET1	PKIB		S100A9	LST1	MERTK	PHLDA1
SERPINF1	PTDSS1	S1RPA		TCF7L2	MS4A7	MS4A4A	PPARG
SLC15A4	SCARB1	TIMM13			MTSS1	NRP1	RGCC
SOX4	TNFRSF10B				RHOC	PA2G4	SDC4
SPIB	XCR1				SERPINA1	PRDM1	SPP1
TCF4					SIGLEC10	SIRPA	TNS3
					SLC44A2	SLAMF8	VEGFA
					TCF7L2	SLCO2B1	
						TREM2	

Table S1C. Signature Genes Used to Define CD4+ T cells subsets. Related to Figure 4.

CD4.c01(CCR7+ Tn)	CD4.c02(TCF7+ Tm)	CD4.c03(CD69+ Tm)	CD4.c04(Temra+ CX3CR1)	CD4.c05(GZMK+ Tem)	CD4.c06(IFNG+ Tfh/Th1)	CD4.c07(IL6ST+ Tfh)	CD4.c08(TNFRSF9+ Treg)	CD4.c09(ISG+ Treg)
ACTN1	ANXA1	ACTB	C1orf21	AOAH	BCL6	BCL6	BATF	BATF
CCR7	ANXA2	CAPG	CCL3	CCL3	BHLHE40	BTLA	CARD16	CCR8
EEF1A1	AREG	CCL4	CCL4	CCL3L3	BTLA	CD200	CCL22	CD70
EEF1B2	CCR7	CCL5	CCL5	CCL4	CCL3	CD40LG	CCR8	CD74
FHIT	CD40LG	CCR2	CMKLR1	CCL4L2	CCL4	CPM	CD70	CSF1
GIMAP7	CD55	CD40LG	CTSW	CCL5	CCL4L2	CXCL13	CD74	ETV7
GPR183	CXCR4	CD52	CX3CR1	CCR5	CCL5	CXCR5	CSF1	FOXP3
IL7R	EEF1A1	CD69	CXCR2	CD4	CCR5	FKBP5	CTLA4	HERC5
KLF2	EEF1B2	CD99	EOMES	CD74	CD200	GNG4	EBI3	IFI6
LEF1	GPR183	CISH	FASLG	CRTAM	CD4	IGFL2	FOXP3	IFIT1
MAL	ICAM2	CKLF	FGFBP2	CST7	CD74	IL21	IKZF2	IFIT3
PLAC8	IL7R	CXCR3	GNLY	CXCR3	CSF2	IL6R	IKZF4	IKZF2
S1PR1	KLF2	EGR1	GZMA	CXCR4	CTLA4	IL6ST	IL1R2	IL1R2
SCML1	KLF3	FASLG	GZMB	DTHD1	CXCL13	ITM2A	IL1RN	IL1RN
SELL	PABPC1	GZMA	GZMH	ENC1	CXCR3	LHFP	IL2RA	IL21R
TCF7	PLAC8	HOPX	GZMK	EOMES	CXCR6	LIF	IL2RB	IL2RA
TPT1	PTGER2	ID2	GZMM	F2R	DUSP4	NMB	IL32	IL2RB
TXK	S1PR1	IFNG	HOPX	FASLG	EOMES	NR3C1	IL7	IL32
	TCF7	KLF2	IL18RAP	GZMA	FAM3C	PDE7B	LAIR2	IL7
	TPT1	LCP2	IL5RA	GZMB	GNG4	RBPJ	LAYN	IRF7
	ZFP36L2	MYADM	KLRD1	GZMH	GZMA	TNFSF11	LTA	ISG15
		MYL12A	KLRG1	GZMK	GZMB	TNFSF4	LTB	LAMP3
		NR4A1	NKG7	IFNG	GZMK	TNFSF8	NAMPT	LAYN
		NR4A2	PLEK	ITM2C	HAVCR2	TOX	PMAIP1	LTA
		PTGER4	PRF1	LITAF	ID2	TOX2	RTKN2	LTB
		RGS1	PRSS23	NFATC2	IFNG	TSHZ2	TBC1D4	MX1
		RGS2	S1PR5	NKG7	IL21	ZBED2	TIGIT	NAMPT
		SH3BGRL3	TBX21	NR4A2	IL2RG		TNFRSF18	OAS1
		SIT1	XCL2	RUNX3	IL6ST		TNFRSF4	RSAD2
		TNF	ZEB2	SLAMF7	KRT86		TNFRSF9	RTKN2
			ZNF683	TNFSF9	LAG3		ZBTB32	STAT1
					MYO7A			TNFRSF18
					NKG7			TNFRSF9
					PDCD1			TNFSF10
					PDE7B			TNFSF13B
					PTMS			USP18
					RBPJ			
					RDH10			
					TIGIT			
					TOX			
					ZBED2			
					ZEB2			

Table S1D. Signature Genes Used to Define CD8+ T cells subsets. Related to Figure 5.

CD8.c01(TCF7+ Tnaive)	CD8.c02(IL7R+ Tm)	CD8.c03(KIR+ NKlike)	CD8.c04(Pre Tex)	CD8.c05(GZMK+ Tex)	CD8.c06(Teminal Tex)	CD8.c07(TCF7+ Tex)	CD8.c08(ISG+ CD8T)	CD8.c09(MKI67+ Prolif)
ACTN1	ACTB	CD160	APOBEC3G	CCL3	CCL3	BATF	CCR1	ACTB
BACH2	ANXA1	CD74	CCL4	CCL3L3	CD70	BTLA	CD74	ACTG1
CCR7	BTG1	CMC1	CCL5	CCL4	CD74	CCR4	CMPK2	CD74
CMTM8	CAPG	CST7	CCR5	CCL5	CSF1	CCR7	IFI44L	CDK1
EEF1A1	CCL5	CXCR4	CD74	CCR5	CTLA4	CD200	IFI6	CENPA
EEF1B2	CD44	DUSP2	COTL1	CD27	CXCL13	CD40LG	IFIT1	CKLF
FLT3LG	CD52	EOMES	CST7	CD74	CXCR6	CPM	IFIT3	CXCR3
IFNGR2	CD55	GCSAM	CXCR3	CST7	DUSP4	CXCL13	IRF7	CYC1
IL23A	CD69	GNLY	EOMES	CTLA4	ENTPD1	EBI3	ISG15	CYCS
IL6R	CISH	GZMA	GZMA	CXCL13	ETV1	GNG4	MX1	E2F1
IL7R	CKLF	GZMB	GZMB	CXCR6	FAM3C	IFNAR2	OAS1	ENO1
KLF2	CX3CR1	GZMK	GZMH	DUSP4	FASLG	IGFBP4	OAS3	GAPDH
LEF1	CXCR4	GZMM	GZMK	EOMES	GEM	IGFL2	PLSCR1	GPI
LTB	DKK3	IFNG	HLA-DPA1	GNLY	GZMB	IL6R	RSAD2	GTF3A
MAL	FTH1	IFNGR1	HLA-DPB1	GZMA	HAVCR2	LAG3	SP100	HMGA1
NELL2	GPR183	IKZF2	HLA-DQA1	GZMB	IFNG	LHFP	STAT1	HMGB1
NOSIP	GZMA	IL2RB	HLA-DQB1	GZMK	IL2RA	NMB	STAT2	KIF20A
PABPC1	IL32	KIR2DL3	HLA-DRB1	HAVCR2	IL2RB	NR3C1	TNFSF10	KIFC1
S1PR1	IL7R	KIR3DL2	HLA-DRB5	HLA-DQA1	IL2RG	SESN3		MIF
SELL	LMNA	KLRD1	IL32	HLA-DRB1	KRT86	TCF7		MKI67
TCF7	MYL12A	KLRF1	ITM2C	IFNG	LAG3	TNFRSF4		MND1
TIMP1	PABPC1	KLRG1	LAG3	LAG3	LAYN	TNFSF8		MXD3
TNFSF8	PFN1	LITAF	SUB1	LYST	NAMPT	TOX		MYB
TPT1	PTPRCAP	NKG7		NAMPT	PDCD1	TSHZ2		NME1
TRABD2A	RGCC	NR4A2		NKG7	RBPJ			NME2
TXK	RPS19	PRF1		PDCD1	TIGIT			PFN1
WNT7A	S100A4	TNFSF9		PRDM1	TNFRSF18			SKA3
	SH3BGRL3	TYROBP		PRF1	TNFRSF9			SPC24
	SIT1	XCL1		TNFRSF9	TNFSF4			TOP2A
	TNF	XCL2		TNFSF4	TOX			
	TNFAIP3	ZNF331		TOX	TOX2			
	TRAF3IP3			TRPS1	ZBED2			
	TSC22D3			TSC22D1				
	ZFP36			VCAM1				
	ZFP36L2							
	ZNF683							

Table S2. Signature Genes for GSVA Analysis. Related to Figure 2 and Figure 5.

Note that this table contains the following worksheets:	
**Worksheet**	**Title**
A	Signature Genes Used to Analyse Cell Cycle, DNA Replication, p53 Signal Pathway Enrichment Scores.
B	Signature Genes Used to Analyse Exhaustion and Effector Scores.

Table S2A. Signature Genes Used to Analyse Cell Cycle, DNA Replication, p53 Signal Pathway Enrichment Scores. Related to Figure 2.

Cell Cycle A2:C69	DNA replication	p53 signal pathway
ANAPC11	AIFM2	DNA2
ANAPC7	CASP3	MCM3
BUB3	CCNE1	MCM7
CCND2	CDK4	POLD2
CDC23	PMAIP1	POLE3
CDC6	TP53	RFC2
CDK2	BAX	RNASEH1
CHEK1	CCNB1	RPA3
E2F3	CCNE2	FEN1
KNL1	CHEK1	MCM4
MCM2	PTEN	PCNA
MCM6	TP53I3	POLD3
ORC1	BCL2L1	PRIM1
PKMYT1	CCNB2	RFC3
RBL1	CDK1	RNASEH2A
SMC3	EI24	SSBP1
TTK	RRM2	LIG1
ANAPC13	BID	MCM5
AURKB	CCND2	POLA2
CCNA2	CDK2	POLE
CCNE1	GTSE1	PRIM2
CDC25A	SIVA1	RFC4
CDC7		RNASEH2C
CDK4		MCM2
DBF4B		MCM6
ESCO2		POLD1
MAD2L1		POLE2
MCM3		RFC1
MCM7		RFC5
ORC5		RPA1
PLK1		
RBX1		
TFDP1		
YWHAE		
ANAPC15		
BUB1		
CCNB1		
CCNE2		
CDC25C		
CDCA5		
CDKN2C		
DDX11		
ESPL1		
MAD2L1BP		
MCM4		
MTBP		
ORC6		
PRKDC		
SGO1		
TP53		
YWHAG		
ANAPC5		
BUB1B		
CCNB2		
CDC20		
CDC45		
CDK1		
CDT1		
E2F1		
FBXO5		
MAD2L2		
MCM5		
NDC80		
PCNA		
PTTG1		
SMC1A		
TRIP13		

Table S2B. Signature Genes Used to Analyse Exhaustion and Effector Scores. Related to Figure5.

Exhaustion	Effector
PDCD1	GNLY
TOX	GZMB
CXCL13	PRF1
TIGIT	IFNG
CTLA4	NKG7
TNFRSF9	GZMA
HAVCR2	GZMK
LAG3	CST7
	TNF
	FASL
	TBX21
